# Influence of Surface Properties on the Adhesion of *Staphylococcus epidermidis* to Acrylic and Silicone

**DOI:** 10.1155/2009/718017

**Published:** 2009-01-25

**Authors:** Cláudia Sousa, Pilar Teixeira, Rosário Oliveira

**Affiliations:** Institute for Biotechnology and Bioengineering (IBB), Centre of Biological Engineering, University of Minho, Campus de Gualtar, 4710-057 Braga, Portugal

## Abstract

The aim of the present study was to compare the ability of eight *Staphylococcus epidermidis* strains to adhere to acrylic and silicone, two polymers normally used in medical devices manufacture. Furthermore, it was tried to correlate that with the surface properties of substrata and cells. Therefore, hydrophobicity and surface tension components were calculated through contact angle measurements. Surface roughness of substrata was also assessed by atomic force microscopy (AFM). No relationship was found between microbial surface hydrophobicity and adhesion capability. Nevertheless, *Staphylococcus epidermidis* IE214 showed very unique adhesion behaviour, with cells highly aggregated between them, which is a consequence of their specific surface features. All strains, determined as being hydrophilic, adhered at a higher extent to silicone than to acrylic, most likely due to its more hydrophobic character and higher roughness. This demonstrates the importance of biomaterial surface characteristics for bacterial adhesion.

## 1. Introduction

*Staphylococcus epidermidis* is a coagulase-negative *staphylococcus* (CNS) that often colonizes the skin and mucous membranes of the human body, representing an important part of its normal microflora [1, 2]. However, these staphylococci have emerged in the last years as the most frequently isolated pathogen in nosocomial sepsis, associated with implanted medical devices [3, 4], namely, prosthetic heart valves and joints, central venous catheters, urinary catheters, contact lenses, and hip prostheses [5]. *S. epidermidis* has the ability to adhere to biomaterials surface and develop as biofilm [6], which constitutes an important virulence factor [7] and the most important pathogenic mechanism of staphylococcal infection [8]. Therefore, initial adhesion of bacteria to the biomaterial surface is thought to be a key step in the colonization of indwelling medical devices. It is a complex process, affected by numerous aspects, such as surface properties of bacteria, material surface properties, and environmental factors [9]. The better understanding of these features is of extreme importance for the development of effective adhesion control mechanisms that will ultimately prevent biofilm formation and thus, the infection of medical devices.

During the adhesion process, bacteria firmly adhere to the biomaterial surface through physicochemical interactions [10]. These comprise cell surface hydrophobicity [11, 12] and charge [13] as well as the hydrophobicity, charge, roughness, and chemical composition of the biomaterial surface itself [9]. Surface hydrophobicity, in particular, has been described as one of the most important properties involved in the adhesion phenomenon [14–16]. According to van Oss and Giese [17], in biological systems, hydrophobic interactions are normally the strongest of the long-range noncovalent interactions and can be defined as the attraction among apolar, or slightly polar, cells or other molecules themselves, when immersed in an aqueous solution. Biomaterial surface roughness is another property relevant for the bacterial adhesion process, with the irregularities of the polymeric surfaces normally promoting bacterial adhesion and biofilm accumulation [18, 19]. This is due to the increased surface area and depressions that provide more favourable and additional sites for colonization [20], as such crevices protect bacterial cells from the shear forces [21]. However, the accumulation of bacteria in such locations depends largely on their size, cell dimension, and division mode [22]. In fact, according to some authors [23, 24], a linear relation of bacterial adhesion with surface roughness is not always verified. A small increase in roughness can lead to a significant increase in bacterial adhesion, while a larger increase in roughness can have no significant effect on cellular attachment.

The aim of the present work was to study the ability of eight strains of *S. epidermidis* to adhere to acrylic and to silicone, two materials commonly used in the manufacture of medical devices, in relation to the surface properties of these materials.

## 2. Materials and Methods

### 2.1. Bacterial Strains

Eight *S. epidermidis* strains were studied in this work. *S. epidermidis* 9142 is a known producer of the surface polysaccharide intercellular adhesin (PIA), which was identified as one of the main responsible factors for biofilm formation [25]. The strain *S. epidermidis* 9142-M10 is an isogenic mutant with a transposon inserted in the *ica* locus that encodes the proteins involved in PIA production and thus does not form biofilm. The PIA-positive *S. epidermidis* 1457 was isolated from an infected central venous catheter, while *S. epidermidis* 1457-M10 is a PIA-negative isogenic mutant of *S. epidermidis* 1457, also obtained by insertion of a transposon into the *ica* locus [26]. *S. epidermidis* IE186, *S. epidermidis* IE214 and *S. epidermidis* IE75 were previously isolated from blood of patients with infective endocarditis, while *S. epidermidis* LE7 was isolated from the skin of a healthy individual. All strains were kindly provided by Dr. G. B. Pier, Channing Laboratory, Department of Medicine, Brigham and Women's Hospital, Harvard Medical School, Boston.

### 2.2. Media and Growth Conditions

For all the assays, cells were firstly grown for approximately 36 hours in plates of Tryptic Soy Agar (TSA; Merck, Germany), and then for 24 hours in 15 mL of Tryptic Soy Broth (TSB, Merck), at 37°C under a constant agitation of 120 rpm. After this period, 50 *μ*L of each suspension were transferred into 30 mL of fresh TSB and incubated for 18 hours, under the same conditions. Then, the cells were centrifuged for 5 minutes at 10 500 × g and 4°C, washed twice with a saline solution (0.9% NaCl (Merck) in distilled water) and sonicated (Ultrasonic Processor, Cole-Parmer, Ill, USA) during 10 seconds, with an amplitude of 22% (previously optimized to avoid cell disruption). The cellular suspension was adjusted to a final concentration of approximately 1 × 10^9^ cells/mL, determined by optical density at 640 nm, prior to usage in the adhesion assays.

### 2.3. Substrate Preparation

2 cm × 2 cm squares of commercial acrylic, specifically Poly(methyl methacrylate) (PMMA) (Repsol, Brønderslev, Denmark) and silicone (Leewood Elastomer AB, Sweden), both with 2 mm in thickness, were used as substrata in the adhesion assays. This acrylic is the synthetic polymer of methyl methacrylate, containing small amounts of a UV-absorber and release agent and with a molecular weight in the range 1.2–1.4 million Da. Concerning silicone, it consists mainly of cross-linked polydimethylvinylmethylsiloxanes with the chain length of ca. 200 000 Da, but also of low chain length polymers in the amount up to typically 2% and not exactly defined amount of fumed silica.

Prior to use, the coupons were washed several times with sterile distilled water and let to soak overnight. Next, they were transferred to a new container with sterile distilled water and washed for 5 minutes under agitation, followed by a 30 minutes immersion period in a 70% ethanol/sterile distilled water solution. Finally, the coupons were aseptically and individually washed with ultra-pure sterile water and let to dry overnight at 60°C.

### 2.4. Adhesion Assays

Adhesion assays were performed as previously described [27]. Briefly, the acrylic and silicone squares were placed in 6-well tissue-culture plates containing 4 mL of bacterial suspension (1 × 10^9^ cells/mL) in saline solution (NaCl, 0.9% (w/v)). Initial adhesion to each substrate was allowed to occur for 2 hours at 37°C, in a shaker rotating at 120 rpm. Negative controls were obtained by placing the coupons in a saline solution without bacterial cells. Each coupon was then carefully removed and washed by immersion, in order to remove loosely attached cells. This procedure was gently undertaken and involved their transference to a glass beaker containing 50 mL of distilled water, where they were kept for about 10 seconds. Afterwards, a new transfer was made to an additional beaker with 50 mL of distilled water, followed by a third transfer 10 seconds later. These washing steps were carefully performed in order to remove loosely attached cells. The coupons were then let to dry at 37°C for about 1 hour. All experiments were done in triplicate and repeated in four independent assays.

### 2.5. Total Cell Counts of Adhered Bacteria

The dried coupons were stained with a 4′-6-diamidino-2-phenylindole (DAPI; Sigma-Aldrich, St Louis, Mo, USA) solution (0.1 g/L) during 30 minutes. Subsequently, each coupon was rinsed with distilled water in order to remove excess stain and let to air-dry in the dark for 30 minutes. Adhered cells were visualised under an epifluorescence microscope (Carl Zeiss, Germany) with a filter sensitive to DAPI fluorescence and coupled with a 3CCD video camera. For each coupon, at least 20 images, with an 820 × 560 resolution and 1000× magnification, were taken. Enumeration of adhered cells was performed with automated enumeration software (SigmaScan Pro 5, SPSS Inc., Chicago, Ill, USA) and the results presented as number of adhered cells/cm^2^.

### 2.6. Scanning Electron Microscopy (SEM)

The coupons with adhered bacteria were dehydrated by a 15-minute immersion in increasing ethanol concentration solutions: 10, 25, 40, 50, 60, 70, 80, 90 and 100% (v/v), having then been placed in a sealed desiccator. Samples were then mounted on aluminium stubs with carbon tape, sputter-coated with gold and observed with a Leica Cambridge S-360 scanning electron microscope (Leo, Cambridge, UK). In order to assess the extent of bacterial adhesion in each sample, three fields were used for image analysis. All photographs were taken using a magnification of 3000×.

### 2.7. Substrata and Bacteria Hydrophobicity

Hydrophobicity parameters of substrata and bacteria surface were determined through the sessile drop contact angle technique [28], using an automated contact angle measurement apparatus (OCA 15 Plus; Dataphysics, Germany). Cleaned and dried substratum surfaces were used for determining the hydrophobicity parameters of acrylic and silicone. In the particular case of bacteria, the measurements were performed on bacterial layers deposited on membrane filters [28]. Briefly, a 30 mL suspension of *S. epidermidis* cells, adjusted to a concentration of approximately 1 × 10^9^ cells/mL in saline solution (NaCl, 0.9% (w/v)), was deposited onto a 0.45 *μ*m cellulose filter (Pall-Life Sciences, USA), previously wetted with 10 mL of distilled water. To standardise the moisture content, the filters with the resultant lawn of cells deposited were then let to dry onto Petri dishes containing 1% (w/v) agar (Merck) and 10% (v/v) glycerol (Sigma-Aldrich), for at least 3.5 hours. All measurements (at least 25 determinations for each material and bacterial strain) were performed at room temperature and water, formamide and **α*-*bromonaphtalene, with known surface tension components [29], were used as reference liquids for standardized contact angles measurements.

Contact angle measurements allowed the calculation of substrata and bacteria hydrophobicity parameters, using the van Oss approach [17, 30]. According to it, the absolute degree of hydrophobicity of a given material (i) is defined in terms of the variation of the free energy of interaction (ΔG) between two moieties of that material, when immersed in water (w), that is, ΔG_iwi_. When ΔG_iwi_ is negative, the free energy of interaction between molecules is attractive, existing a smaller affinity for water than among molecules themselves, making (i) (the microbial cell or material surface) hydrophobic. In an opposite way, (i) is hydrophilic when ΔG_iwi_ is positive.

### 2.8. Atomic Force Microscopy (AFM)

Acrylic and silicone surfaces topography was assessed by atomic force microscopy (AFM), using a PicoPlus scanning probe microscope from Molecular Imaging (USA). Surface imaging was performed in Tapping mode and the samples were analysed in air at room temperature. The acrylic surfaces were analysed using a silicon (Si) tip with a Spring Constant ≅42 N/m, while for silicone surfaces the Si tip had a Spring Constant ≅2.8 N/m. The roughness measurements were performed under a scan range of 2.5 × 2.5 *μ*m, using the SPIP version 4.2.2.0 software. Measurements were made in three randomly chosen areas in all samples.

### 2.9. Statistical Analysis

Results from all the assays were compared using one-way analysis of variance (ANOVA) by applying Levene's test of homogeneity of variances and the Tukey multiple comparisons test, using software Statistical Package for the Social Sciences Inc., (SPSS) (Chicago, Ill, USA). All tests were performed with a confidence level of 95%.

## 3. Results

### 3.1. Adhesion to Acrylic and Silicone

**Figure 1:**
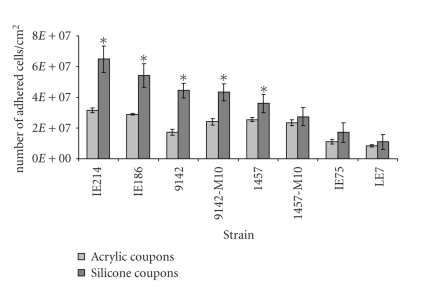
Number of adhered cells per cm^2^ onto acrylic (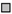) and silicone (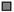) coupons, after a 2-hour period of contact for *S. epidermidis* strains IE214, IE186, 9142, 9142-M10, 1457, 1457-M10, IE75, and LE7. The symbol (∗) indicates the strains that adhered at a statistically higher extent to silicone than to acrylic (*P* < .05).

The initial adhesion of the *S. epidermidis* strains to the acrylic and silicone surfaces is presented in Figure 1. As it can be seen, almost all *S. epidermidis* strains adhered at a significantly (*P* < .05) higher extent to the silicone substrate than to acrylic. The only exceptions were observed for strains 1457-M10, IE75, and LE7, which also adhered more to silicone than to acrylic but in a nonsignificant way (*P* > .05). In fact, the extent of adhesion of *S. epidermidis* 9142 to silicone was approximately 2.5 times greater than to acrylic. For strains 9142-M10, IE186, and IE214, this difference ranged between 1.8 and 2.0 times. Concerning acrylic, strains IE214 and IE186 were the ones that most extensively, and significantly (*P* < .05) adhered to the coupons. In opposition, strains IE75 and LE7 showed the lowest levels of initial binding to this material, being markedly different from all the other strains (*P* < .05). Similarly, they also adhered least to silicone coupons (*P* < .05). *S. epidermidis* IE214 and *S. epidermidis* IE186 were the strains showing the highest number of cells adhered (*P* < .05) to this substrate, likewise to acrylic.

**Figure 2:**
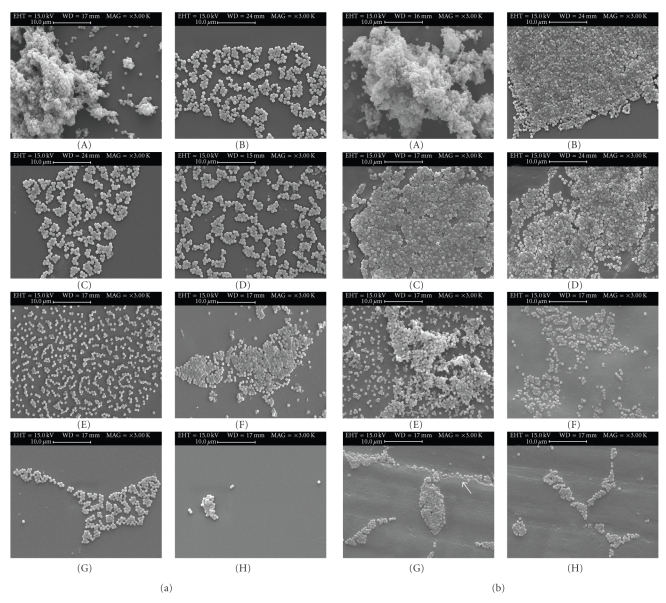
SEM photomicrographs of *S. epidermidis* adhered to acrylic (a) and silicone (b) surfaces. Strains: A—IE214; B—IE186; C—9142; D—9142-M10; E—1457; F—1457-M10; G—IE75; H—LE7. The arrow shows bacterial cells adhered along a depression on silicone's surface. Magnification ×3000, bar = 10 *μ*m.

Scanning electron microscope (SEM) images of bacteria adhered to acrylic (a) and silicone (b) squares are presented in Figure 2. The images reveal grape-like clusters of variable dimensions. It is also visible the higher extent of adhesion to silicone than to acrylic, especially for strains IE214, IE186, 9142, 9142-M10, and 1457. In addition, it can be seen, both for acrylic and for silicone, how strain IE214 cells (Figure 2(a)-A and Figure 2(b)-A) grew highly aggregated, quite differently from the mode of growth of the remaining strains. In fact, this strain formed flocculent suspensions in liquid medium. In Figure 2(b)-G, it must be noted (arrow) how cells adhered to the silicone along the depression on its surface.

### 3.2. Substrata and Bacteria Hydrophobicity

Hydrophobicity of substrata and bacteria was evaluated through contact angle measurements, using the van Oss approach [17, 30]. Contact angles, surface tension parameters, and hydrophobicity of acrylic and silicone are presented in Table 1. Water contact angles can be used as a qualitative indication of the surface material hydrophobicity, with higher values indicating a more hydrophobic surface. As it can be seen, the water contact angles obtained for both surfaces are high, a fact that is indicative of their hydrophobicity. The values of ΔG_iwi_ also showed that both materials are hydrophobic (ΔG_iwi_ <0), with silicone holding a more hydrophobic character. From Table 1, it can also be seen that both acrylic and silicone surfaces are predominantly electron donors (higher values of *γ* ^−^), with low electron acceptor parameters (*γ* ^+^). In fact, acrylic does not have an electron acceptor parameter (*γ* ^+^ = 0) but is only electron donor (*γ* ^−^).

Cell surface hydrophobicity parameters of *S. epidermidis* strains, as well as the contact angles obtained using the three liquids tested, are presented in Table 2. The eight strains studied showed similar values of water contact angles, lower than 65°, which in accordance to Vogler [31] is indicative of a hydrophilic surface, ranging from 21.6° (strain IE186) to 31.8° (*S. epidermidis* 1457). These values are quite similar to those obtained for formamide, also polar, with exception of strain LE7 that presented a formamide contact angle much lower than that of water. The contact angles determined by using the apolar liquid, **α**-bromonaphtalene, showed small variation between strains with values higher than 49.6° (*S. epidermidis* 1457-M10). Also, all strains showed positive values of ΔG_iwi_ and so, can be considered hydrophilic. In what concerns surface tension components, all strains predominantly showed electron donation, with higher values of electron donor parameters (*γ* ^−^) compared to the low values of the electron acceptor parameters (*γ* ^+^). Strain IE214 showed the highest values of electron acceptor and electron donor parameters of the acid-base component of the surface tension, while strains IE186 and LE7 revealed the lowest values of electron acceptor and electron donor parameters, respectively.

### 3.3. Substrata Roughness

**Figure 3:**
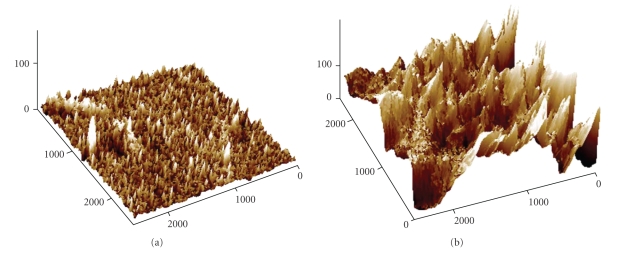
AFM images of acrylic (a) and silicone (b) surfaces with a scan range of 2.5 *μ*m × 2.5 *μ*m (air Tapping mode). Axis *x* and *y*-nm; axis *z*−Å.

The surface topography of acrylic and silicone was analyzed by AFM in tapping mode (Figure 3). Silicone surface displays higher roughness with an average value (Ra) of 4.237 nm and a maximum (Rmax) of 44.367 nm, in opposition to Ra = 0.789 nm and Rmax = 15.683 nm for acrylic surface. Ra indicates the average distance of the roughness profile to the centre plane of the profile, while Rmax represents the maximum height measured within the screened area.

## 4. Discussion

*S. epidermidis* is strongly associated with infections related to implants and medical devices, such as joint prosthesis, prosthetic heart valves, vascular catheters and contact lenses [3, 32, 33]. Given the fact that acrylic and silicone are materials normally used in the production of some of these devices, it is of major importance to study the adhesion of *S. epidermidis* to these polymers. Thus, the primary intention of this study was to attempt to correlate the adhesion capability of 8 *S. epidermidis* strains with the hydrophobicity parameters of cells and these materials surfaces. Surface roughness of acrylic and silicone was also assessed.

As the present results indicated, all *S. epidermidis* strains adhered at a higher extent to silicone substrate than to acrylic (Figure 1). These results are in accordance with other studies that refer silicone rubber as being especially prone to being colonized by *Candida*, streptococci, *Pseudomonas* species, and also by staphylococci and other CNS, depending on the site of implantation [34–36]. Taking into consideration water contact angle values (114.5 ± 2.3°) and the hydrophobicity degree parameter, ΔG_iwi_ = −67.1 mJ/m^2^, the silicone assayed was found to be more hydrophobic than acrylic (water contact angle = 85.3 ± 2.2°; ΔG_iwi_ = −62.5 mJ/m^2^) (Table 1), despite this difference was more pronounced if only water contact angles values were considered. These results are in accordance with values previously obtained [12, 37] and clearly demonstrate the importance of the material hydrophobic effect in initial adhesion, since acrylic and silicone have both a hydrophobic character. A higher surface hydrophobicity of silicone is probably responsible for the highest levels of initial binding to this substrate. This fact is corroborated by the work of Oliveira et al. [12] where the attachment of *S. epidermidis* to four polymeric materials (including silicone), commonly used in indwelling medical devices, was assayed. All materials were considered hydrophobic (ΔG_iwi_ <0) and an increase in the degree of hydrophobicity was linearly correlated with the number of attached cells. A point also to be noted is that acrylic stands solely as an electron-donor (*γ* ^−^). Given the fact that a microorganism may adhere to a substratum via the hydrophobic effect, that is, if hydrophobic areas are available for interactions with hydrophobic sites on substrata [38], the lower densities of apolar areas in acrylic (*γ* ^+^ = 0) help to justify the preferential adhesion of *S. epidermidis* cells to a more hydrophobic material such as silicone.

In what concerns cell surface hydrophobicity parameters determined, all strains were considered to be hydrophilic (ΔG_iwi_ >0) (Table 2). The most hydrophilic *S. epidermidis* strain, IE186, (ΔG_iwi_ = 32.5 mJ/m^2^) was the second most adherent strain to both materials, while the least adherent, strain LE7, was the one with the weakest hydrophilic character. Thus, contrary to what was found for materials hydrophobicity, no correlation was found between cell surface hydrophobicity of the *S. epidermidis* strains and their ability to adhere to both hydrophobic surfaces. This fact is corroborated by previous studies [37, 39] and suggests that other cell surface factors can as well contribute to the initial attachment to biomaterials surfaces, such as the production of exopolysaccharides like extracellular polysaccharide adhesins and autolysins with adhesive properties like AtlE [40] and Aae [41]. In fact, some authors attribute cell surface hydrophobicity to covalently bound cell-wall-associated proteins [42–45]. Furthermore, it was observed (Table 2) that all cell surfaces were predominantly electron donors (higher values of *γ* ^−^), with low electron acceptor parameters (*γ* ^+^). This polar character can be due to the presence of residual water of hydration or polar groups [46]. However, the high value of the electron acceptor parameter of strain IE214 can justify its highest number of cells adhered to both materials by increasing the interactions between the electron-donor groups of the substrata and the electron-acceptor groups of cells. These slightly higher values of surface tension parameters comparing to the other strains can also indicate that acid-base interactions between cells of this strain are more favoured than in the other *S. epidermidis* strains studied. Therefore, when the first *S. epidermidis* IE214 cells approach the surface, they have good conditions to adhere to it, but as long as more cells approximate, they tend to adhere to another close cell instead to the surface itself. This leads to the formation of prominent cell aggregates that can be seen in Figure 2, which were still firmly adhered to the surface. The polysaccharide intercellular adhesin (PIA), a polymer of *N*-acetyl glucosamine [25] synthesised by enzymes encoded by the *ica* operon [47], is crucial to the cell-to-cell adhesion process and biofilm accumulation [48, 49]. According to the hemagglutination ability [37], which reflects the level of PIA expression, *S. epidermidis* IE214 is a strong producer of PIA (hemagglutination titer of 1:16). Therefore, the high levels of PIA production by *S. epidermidis* IE214, along with its physicochemical properties, namely, surface tension, aid to support the unique behaviour of *S. epidermidis* IE214, compared to the remaining strains. These specific physicochemical properties are most probably due to IE214 being the only strain capable of producing a 148 kDa cell wall protein (AtlE) (data not shown), which has been described as determinant in the adhesion to unmodified polymer surfaces [40].

In addition to hydrophobicity and surface tension parameters, the material surface roughness is another factor that has been pointed out as capable of influencing bacterial adhesion to a given material [50]. This is probably due to the fact that rough surfaces have greater surface areas and that depressions in the roughened surfaces provide more favourable sites for colonisation [51]. In fact, according to van Hoogmoed et al. [52], there is a microorganism's preference for adherence to scratches or grooves, which could be seen in Figure 2(b)-G. The AFM results obtained showed that the average roughness is higher for silicone than for acrylic. However, it is difficult to ascertain the possible effect of this parameter in cocci adhesion, since for both surfaces the roughness is at a nanoscale, meaning that there are no microcrevices in the surfaces to act as niches for the microbial cells. Nevertheless, according to Katainen et al. [53], while in particles smaller than the surface features the interaction is limited to one contact between the particle and a single asperity, being the strength of adhesion determined by this only contact, particles larger than the surfaces features (which is the present case for silicone) have several contacts with the surface. This thus allows a higher level of interaction leading to a major influence on the adhesion phenomenon.

Therefore, a stable bacterial adhesion to a biomaterial requires a high degree of hydrophobicity, as well as a certain degree of roughness between other physicochemical properties of the substratum [10]. Silicone is widely used as a biomaterial but it has the disadvantages of being more hydrophobic and rougher than acrylic, thus becoming a more prone material to *S. epidermidis* adherence.

## 5. Conclusions

Bacterial adhesion to the less hydrophobic material (acrylic) was significantly lower than to the more hydrophobic (silicone). These results showed the importance of the hydrophobic effect of the biomaterial surface in initial adhesion. The higher roughness of silicone seems also to exert some effect in bacterial adhesion. On the other hand, bacteria surface physicochemical properties seem to have less effect in their binding to substrata, highlighting the importance of other cell surface factors to the initial adhesion process. Nevertheless, *S. epidermidis* strain IE214 revealed completely distinct adherence behaviour compared to the remaining strains, probably as a consequence of its unique surface features, as displayed by its flocculence ability.

## Figures and Tables

**Table 1:** Water (*θ*_*W*_), formamide (*θ*_*F*_), and **α**-bromonaphtalene (*θ*_*α*−*B*_) contact angles (in degrees), surface tension components, and hydrophobicity (in mJ/m^2^) of the acrylic and silicone coupons surface.

Substratum	Contact angle ± SD (°)			Surface tension components (mJ/m^2^)			ΔG_iwi_ (mJ/m^2^)
	*θ*_*W*_	*θ*_*F*_	*θ*_*α*−*B*_	*γ* ^LW^	*γ* ^+^	*γ* ^−^	
Acrylic	85.3 ± 2.2	64.1 ± 1.2	24.5 ± 1.2	40.5	0.0	4.5	−62.5
Silicone	114.5 ± 2.3	104.3 ± 2.4	81.4 ± 3.5	14.7	0.4	1.7	−67.1

SD: standard deviation; *γ* ^LW^: apolar Lifshitz-van der Waals surface free energy component; *γ* ^+^: electron acceptor surface free energy component; *γ* ^−^: electron donor surface free energy component; ΔG_iwi_: degree of hydrophobicity.

**Table 2:** Water (*θ*_*W*_), formamide (*θ*_*F*_), and *α*-bromonaphtalene (*θ*_*α*−*B*_) contact angles (in degrees), surface tension components, and hydrophobicity (in mJ/m^2^) of the surface of *S. epidermidis* strains.

*S. epidermidis* strain	Contact angle ± SD (°)			Surface tension components (mJ/m^2^)			ΔG_iwi_ (mJ/m^2^)
	*θ*_*W*_	*θ*_*F*_	*θ*_*α*−*B*_	*γ* ^LW^	*γ* ^+^	*γ* ^−^	
IE214	22.3 ± 3.5	21 ± 1.2	59 ± 2.1	20.6	7.9	56.7	20.3
IE186	21.6 ± 1.6	29.9 ± 4.0	54.5 ± 2.0	27.7	2.5	55.5	32.5
9142	25.6 ± 0.9	25.4 ± 2.6	57.0 ± 1.4	26.5	4.0	48.4	22.8
9142-M10	21.8 ± 1.2	19.0 ± 1.9	54.7 ± 1.0	27.6	4.3	48.4	22.0
1457	31.8 ± 1.0	31.4 ± 2.5	53.2 ± 1.5	28.4	2.7	45.9	22.8
1457-M10	24.7 ± 1.8	17.3 ± 0.7	49.6 ± 0.9	30.1	3.8	45.3	19.6
IE75	27.1 ± 1.0	26.5 ± 1.6	50.4 ± 1.3	29.8	2.7	47.9	24.2
LE7	23.7 ± 0.7	9.4 ± 0.6	52.3 ± 1.3	28.8	5.0	43.6	16.5

SD: standard deviation; *γ* ^LW^: apolar Lifshitz-van der Waals surface free energy component; *γ* ^+^: electron acceptor surface free energy component; *γ* ^−^: electron donor surface free energy component; ΔG_iwi_: degree of hydrophobicity.
